# Fermented medicinal herbs improve the hematological and physiological profile of Striped catfish *(Pangasi* *onodon* *hypophthalmus)*

**DOI:** 10.12688/f1000research.52640.1

**Published:** 2021-06-14

**Authors:** Henni Syawal, Ronal Kurniawan, Irwan Effendi, Brian Austin

**Affiliations:** 1 Universitas Riau, Pekanbaru, 28294, Indonesia; 2 Insitute of Aquaculture, University of Stirling, UK, UK

**Keywords:** Medicinal herb, Catfish, Immunostimulant, Hematology, Physiology, Immunity

## Abstract

This study sought to determine the effect of fermented medicinal herbs (FMH), i.e. cutchery ( *Kaempferia galanga*), tumeric ( *Curcuma longa*) and curcuma ( *Curcuma xanthorrhiza*) in combination with molasses and probiotic drink (Yakult), administered orally on the hematological and physiological profile of striped catfish ( *Pangasionodon hypophthalmus*). A complete randomized design (CRD) experiment was used with four levels of treatments, namely P0 (control), P1 (FMH 100 ml/kg), P2 (FMH 200 ml/kg) and P3 (FMH 300 ml/kg) of feed. The fish were kept in a farm in cages at 75 fish/m ^3^_, _and fed with the experimental diets for 60 days. The results revealed that FMH (P2) dietary administration improved hematological and physiological profile of catfish, i.e total erythrocytes of 2.81 x 10 ^6^ cells/mm ^3^, hematocrit values of 39.00 %, hemoglobin levels of 10.73 g/dl, total leukocytes of 11.41 x 10 ^4^ cells/mm ^3^, blood glucose 97.33 mg/dl, and total serum protein 4.10 mg/dl compared to controls with 1.89 x 10 ^6^ cells/mm ^3^, 32.33 %, g/dl, 9.67 x 10 ^4^ cells/mm ^3^, 67.33 mg/dl, and total serum protein of 3.10 mg/dl, respectively. Moreover, the diet improved special growth rate, feed conversion ratio, feed efficiency and the survival rate of catfish

## Introduction

Attention in aquaculture in developing countries has focused on the use of nonspecific immunostimulants and plant products, which could have a beneficial effect in fish disease control. Interest in medicinal plants for application to aquaculture follows their use in human medicine and agriculture as proven prophylactic and therapeutic agents. ^1^^–^^3^ For example, garlic ( *Allium sativum*) and ginger ( *Zingiber* officinale) ^4^ have histories of dietary and medicinal applications as anti-infective agents. Evidence of their value include inhibition towards pathogens of relevance to aquaculture, including bacteria, ^5^^–^^7^ viruses, ^8^^–^^10^ and protozoa. ^11^^–^^12^

Some researchers have reported the impact of herbal supplemented diets on hematology and innate immunity of fish. ^13^^–^^14^ For example, Chinese herbs ( *Lonicera japonica* and *Ganoderma lucidum*) enhanced the non-specific immune response of tilapia ( *Oreochromis niloticus),* leading to protection against *Aeromonas hydrophila*. ^15^ Similarly, the dietary administration of rose hip and safflower stimulated growth performance, hematological, biochemical parameters and innate immune responses of beluga ( *Huso huso*) ^16^^,^^17^ The supplemented probiotic stimulated growth performance and feed utilization of keureling fish *Tor tambra.* Against this background, fermented medicinal herbs (FMH) in combination with molasses and probiotic drink (Yakult) have been used successfully as dietary supplements on a small number of rural catfish farms in Sumatra, Indonesia, for approximately two years. Anecdotal evidence has suggested that fish grow better and faster, appear to be healthier, and the flesh tastes better after cooking. Fish, which have received these diets, have been examined for potential improvements to hematology and physiology.

## Methods

### Experimental diet

This research was conducted on floating net cages in the Reservoir of the Faculty of Fisheries and Marine Affairs by using a completely randomized design with four treatments and three replications, namely P0 (control), P1 (FMH 100 mL/kg feed), P2 (FMH 200 mL/kg), and P3 (FMH 300 mL/kg). all steps in this experiment was carried out within the international ethical guidelines provided by ARRIVE guidelines.

### Fish

A total of 2700 fingerling catfish used had an average length of 9 cm and an average weight of 6 g. Catfish were obtained from local farmers in Kampar Regency and distributed randomly into cages measuring 1 × 1.5 × 1 m with a density of 75 individuals/m ^3^. The cages were constructed of polyethylene nets with a mesh size of 7 × 7 mm. Water was free-flowing, and the temperature ranged 27.5–29.5°C.

All fish were acclimatized for seven days before use. The fish samples were selected based on their performance. The fish that showed active swimming, no wounds or external parasites. To measure the growth of the fish, every 10 days a random weighing of the fish was carried out.

### Use of dietary supplements

The dietary supplements were chosen because of their availability, and use in local, Sumatran, aquaculture. Thus, fermented medicinal herbs (FMH) consisting of cutchery ( *Kaempferia galanga*), turmeric ( *Curcuma longa*) and curcuma ( *Curcuma zanthorrhiza*) together with molasses, probiotic drink (Yakult), fresh water and yeast ( *Saccharomyces cerevisiae*) were used ( Table 1). Fresh turmeric, cutchery, and curcuma were washed, thinly sliced, mixed and milled. Then, 300-g quantities were transferred to 3 L of water, boiled for 30 min, and cooled to room temperature. The mixture was then squeezed and filtered to obtain the liquid fraction, which totalled 2.7 L. This was mixed with 175 mL of molasses, 65 mL of probiotic drink and 50 g of yeast. Then, the mixture was stirred until homogeneous and poured into 5-L capacity jerry cans, which were tightly closed. Fermentation was allowed to occur for 7–10 days until the aroma changed from a curcuma smell to a strong alcoholic odour, and gas was no longer produced. The gas produced during fermentation was released daily by opening the lids for a few seconds. FMH was added to 1-kg quantities of pelleted feed (Hi-Pro-Vite 781 PT. Central Proteina Prima Tbk) to achieve 0 (control; P0), 100 mL/kg (P1), 200 mL/kg (P2) and 300 mL/kg (P3) The feed was administered three times a day to achieve 5% of body weight for 60 days, which reflected the period that the diets have been used on fish farms. Survival rates were calculated using the following formula, SR = Nt/No×100%, where SR = survival (%), Nt = number of live fish at the end of the study, and No = number of live fish at the beginning of the study.

**Table 1.** Composition of medicinal herb mixture.

Items	Amount
Fresh tumeric rhizome (g)	100
Fresh cutchery rhizome (g)	100
Fresh curcuma rhizome (g)	100
Molasses (mL)	175
Probiotic drink (mL)	65
Mineral water (mL)	3000
Yeast (mg)	50

### Examination of fish blood

Fish blood was removed at 0, 30 days and 60 days. Thus, the fish were anesthetized with clove oil at a dose of 0.05 mL/L. Blood was withdrawn from the caudal vein using 1-mL syringes that had been rinsed with 10% EDTA, collected in microtubes (Axygon) and stored at room temperature until use. The fish that have been blood drawn are then awakened by placing them in a container filled with aerated water. The fish were returned to the cages after they were seen breathing and swimming normally.

To determine the number of erythrocytes, blood in 0.1-mL quantities was mixed thoroughly with 1.0 mL of Hayem solution comprising sodium sulfate, sodium chloride and mercuric chloride. ^19^ Then, the number of erythrocytes was determined by use of a hemocytometer at a magnification of ×40 with calculation according to the formula of Blaxhall and Daisley. ^19^ The method of Blaxhall and Daisley ^19^ was used to estimate the total number and types ( *i.e.* lymphocytes, monocytes, neutrophils, and platelets) of leucocytes. For this, blood was dripped onto a hemocytometer slide, covered with a coverslip, and examined microscopically with a magnification of ×40. The total number of leukocytes was calculated using the following formula: ∑Leukocytes = ∑n × 50 cells/mm ^3^, where ∑n = total number of leukocytes in four large boxes, and 50=dilution factor. Calculation of hemoglobin levels was carried out using Sahli’s method. ^20^ To determine hematocrit level, blood was drawn into a hematocrit capillary tube the end of which was blocked with crystoseal. ^19^ Then the capillary tube was centrifuged for 3 min at 11000 rpm in a microhematocrit centrifuge Model SH120-1. The hematocrit was measured as a percentage of the hematocrit value in the microhematocrit reader

Blood glucose was measured using GlucoDr (allmedicus) with a range of 20–600 mg/dL. Glucose testing was carried out in the morning before the fish were fed. ^21^ Total serum protein was measured by the method of Anderson and Siwicki. ^22^ Blood was centrifuged at 3,500 rpm for 15 min to completely separate the serum, which was transferred to a fresh microtube. Then, 20 μL of serum and 1000 μL of protein test reagent (Reiged Diagnostics) were added to each microtube, with mixing. After incubation for 15 min, the absorbance was read at λ 595–610 nm.

### Growth parameters

a) The absolute weight was measured using the formula **AW = Wt − Wo**, where AW = absolute weight (g), Wt = average weight at the end of the study (g), and Wo = average length at the beginning of the study (g).
b) Specific growth rates were calculated using the formula, **SGR =** $\mathrm{SGR}=\frac{\mathrm{Wt}-\mathrm{Wo}}{t}\times 100$ , where Wt = larvae weights at the end of the study (g), Wo = larvae weights at the beginning of the study (g), and t = length of study (day).
c) Feed conversion was calculated by using the formula, $\mathrm{FCR}=\frac{\mathrm{\Sigma F}}{\left(\mathrm{Bt}+\mathrm{Bm}\right)-\mathrm{Bo}}$ , where FCR = feed conversion ratio, ΣF = amount of feed fed during experiment (g), Bt = fish biomass weight at the end of maintenance (g), Bo = fish biomass at the beginning of study (g), and Bm = biomass of dead fish during maintenance (g).
d) Feed efficiency was calculated by using formula, **FE = (Bt + Bm) − Bo)/F × 100**, where FE = feed efficiency, ΣF = amount of feed fed during experiment (g), Bt = fish biomass at the end of experiment (g), Bm = biomass of fish that died during the study (g), and Bo = fish biomass at the beginning of the study (g).

### Statistical methods

The experiments employed a completely randomized design (CRD) involving use of SPSS version 22.

### Statement on animal ethics

All animals are treated according to animal welfare guidelines that have been established and approved by the Dean of the Faculty of Fisheries and Marine Sciences, Riau University (Prof. Bintal Amin, serves as the ethical committee who approved the use of vertebrate animals in this experiments with number: 346/UN19.5.1.1.4/KP/2020).

## Results

The fish consumed the experimental diets better than the controls without any evidence of a period of adjustment. Moreover, compared with the controls, the fish receiving the experimental diets were more active, responding quickly to the arrival of the feed. Overall, the FMH diets improved the growth rate of fish significantly ( *p* < 0.05). In addition, the provision of the supplements maintained the survival rate of catfish at 100% ( Table 2).

**Table 2.** Growth of striped catfish during the study.

Treatment	Parameters				
	Absolute weight (g)	SGR (%/day)	FCR	Feed efficiency (%)	SR (%)
P0	89.11 ± 3.71 ^a^	4.45 ± 0.05 ^a^	1.57 ± 0.03 ^d^	63.81 ± 1.51 ^a^	96
P1	108.95 ± 0.09 ^b^	4.72 ± 0.03 ^b^	1.35 ± 0.01 ^c^	73.87 ± 0.30 ^b^	100
P2	119.08 ± 1.02 ^c^	4.86 ± 0.02 ^c^	1.21 ± 0.01 ^a^	82.93 ± 0.42 ^d^	100
P3	112.02 ± 1.18 ^b^	4.77 ± 0.05 ^b^	1.25 ± 0.01 ^b^	79.99 ± 0.60 ^c^	100

Note: Superscript letters on the same line show significantly different results between treatments ( *p* < 0.05). SGR: specific growth rate, FCR: feed conversion ratio, SR: survival rate.

FMH diets improved the hematological profile of striped catfish significantly ( *p* < 0.05) compared to the experimental controls. Thus, total erythrocyte counts ranged between 2.45–2.81 × 10 ^6^ cells/mm ^3^, hematocrit values were from 35.67 to 39.00%, and hemoglobin levels ranged from 9.2–10.73 g/dL ( Table 3). This compares with 1.89 × 10 ^6^ cells/mm ^3^, 32.33 %, and 8.80 g/dL, for the controls ( Table 3). Consistently, diet P2 exhibited the best level of hematological profiles at both 30 and 60 days after initiating the feeding regime ( Table 3). Moreover, the examination of the groups revealed that the fish were in excellent condition, were more agile, and appeared to have a better colour. The internal organs were normal in appearance.

**Table 3.** Results of measurement of striped catfish hematological profile during the study. Superscript letters on the same line show significantly different results between treatments ( *p* < 0.05). RBC: red blood cell (×10 ^6^ cells/mm ^3^); WBC: white blood cell (×10 ^4^ cells/mm ^3^).

Treatment	Parameters							
	RBC	Hematocrit (%)	Hemoglobin (g/dL)	WBC	Lymphocyte (%)	Monocyte (%)	Neutrophile (%)	Thrombocyte (%)
Day 30								
P0	1.75 ± 0.03 ^a^	29.00 ± 1.00 ^a^	8.80 ± 0.20 ^a^	9.20 ± 0.05 ^a^	76.67 ± 0.58 ^a^	7.33 ± 0.58	7.67 ± 0.58 ^b^	8.67 ± 0.58 ^b^
P1	1.82 ± 0.01 ^b^	35.00 ± 1.00 ^b^	9.27 ± 0.23 ^b^	9.47 ± 0.11 ^b^	78.67 ± 0.58 ^b^	6.67 ± 0.58	6.33 ± 0.58 ^ab^	8.33 ± 0.58 ^b^
P2	1.91 ± 0.02 ^c^	37.00 ± 1.00 ^b^	10.73 ± 0.11 ^d^	10.65 ± 0.05 ^d^	81.67 ± 0.58 ^c^	6.33 ± 0.58	5.33 ± 0.58 ^a^	6.67 ± 0.58 ^a^
P3	1.85 ± 0.02 ^b^	37.00 ± 1.00 ^b^	9.87 ± 0.11 ^c^	10.31 ± 0.03 ^c^	79.33 ± 0.58 ^b^	6.33 ± 0.58	6.67 ± 0.58 ^ab^	7.67 ± 0.58 ^ab^
Days 60								
P0	1.89 ± 0.07 ^a^	32.33 ± 2.08 ^a^	9.20 ± 0.05 ^a^	9.67 ± 0.14 ^a^	77.33 ± 0.58 ^a^	7.33 ± 0.58	7.33 ± 0.58 ^b^	8.00 ± 1.00 ^b^
P1	2.45 ± 0.10 ^b^	35.67 ± 0.58 ^b^	9.47 ± 0.11 ^b^	10.04 ± 0.09 ^b^	79.67 ± 0.58 ^b^	6.67 ± 1.15	6.67 ± 0.58 ^ab^	7.00 ± 1.00 ^ab^
P2	2.81 ± 0.02 ^c^	39.00 ± 1.00 ^c^	10.65 ± 0.05 ^d^	11.41 ± 0.09 ^d^	82.33 ± 0.58 ^c^	6.00 ± 1.00	6.00 ± 1.00 ^a^	5.33 ± 0.58 ^a^
P3	2.53 ± 0.06 ^b^	38.33 ± 0.58 ^c^	10.31 ± 0.03 ^c^	11.14 ± 0.13 ^c^	80.33 ± 0.58 ^b^	6.33 ± 0.58	6.67 ± 0.58 ^ab^	6.67 ± 0.58 ^ab^

FMH affected the physiological profile of striped catfish significantly ( *p* < 0.05) compared with the controls. Thus, blood glucose levels and total serum protein increased from 78.67 to 97.33 mg/dL and 3.70 to 4.10 mg/dL; osmoregulation ranged from 288–327 mOsm/L H _2_O, absolute weight from 89.11–119.08 g, and the survival rate from 96–100% ( Table 4).

**Table 4.** Physiological profile of striped catfish during the study. Superscript letters on the same line show significantly different results between treatments ( *p* < 0.05). SR: survival rate.

Treatment	Parameters				
	Blood glucose (mg/dL)	Serum total protein (mg/dL)	Osmoregulation (mOsm/L H _2_O)	Absolute weight (g)	SR (%)
P0	67.33 ± 5.03 ^a^	3.10 ± 0.27 ^a^	293 ± 9.86 ^a^	89.11 ± 3.71 ^a^	96
P1	78.67 ± 4.16 ^b^	3.70 ± 0.10 ^b^	289.33 ± 7.51 ^a^	108.95 ± 0.09 ^b^	100
P2	97.33 ± 7.02 ^c^	4.10 ± 0.10 ^c^	289.67 ± 18.50 ^a^	119.08 ± 1.02 ^c^	100
P3	96.67 ± 5.51 ^c^	3.97 ± 0.15 ^bc^	327.00 ± 16.70 ^b^	112.02 ± 1.18 ^b^	100

## Discussion

This study has supported the use of FHM feed supplements in Indonesian aquaculture, and provided evidence for the mode of action. Thus, there were notable improvements in the hematological and physiological profiles of catfish. Indeed, similar results have been reported by other researchers. For example, Lee *et al.* ^23^ evaluated the dietary supplementation of citrus by-products (CB) fermented with probiotic bacteria on growth performance, feed utilization, innate immune responses and disease resistance of juvenile olive flounder. They noted that innate immunity was significantly enhanced by CBF–BS (CB fermented with *Bacillus subtilis*) supplementation. This study indicated that the fermentation of CB with probiotic had beneficial effects on innate immunity and thereby increased disease resistance.

The important components of FMH are considered to be due to the content of secondary metabolites, namely curcuminoids, vitamin C, essential oils, tannins, and flavonoids, which trigger immunostimulation. ^24^ The presence of curcuminoids provide an antioxidant effect on cell membranes reducing erythrocyte cell membrane damage due to oxidation. ^25^ Similarly, flavonoids are natural antioxidants, which are credited with the ability of reducing free radicals and anti-free radicals. ^26^

The benefit of FMH for improved growth performance mirrors previous work by Hoseinifar *et al.*, ^27^ who reported that dietary application of the phytoimmunostimulant Persian hogweed ( *Heracleum persicum*) improved growth significantly. Specifically, the final weight, weight gain, specific growth rate and feed conversion ratio were significantly improved in fish that received dietary *H. persicum* at or above 5 g/kg. ^27^

Clearly, this study has demonstrated that dietary FMH, particularly when dosed at 200 mL/kg of feed, improved the hematological and physiological profile of catfish. Also, there was benefit for specific growth rate, feed conversion ratio and survival.

## Data availaibility

All data underlying the results are available as part of the article and no additional source data are required
