# Supplementary material for: Fermented medicinal herbs improve hematological and physiological profile of Striped catfish (Pangasi anodon hypophthalmus)
Source: F1000Res. 2022 Apr 19;10:466. Originally published 2021 Jun 14. [Version 3] doi: 10.12688/f1000research.52640.3 (PMC9021665; doi:10.12688/f1000research.52640.3)
Supplement: Peer review history

# Peer review: Reviewer response for version 3

**DOI:** 10.5256/f1000research.128325.r135069

**Published:** 2022-04-20

**Authors:** Mohammed El Basuini

**Affiliations:** 1 Department of Animal Production, Faculty of Agriculture, Tanta University, Tanta, Egypt

The authors have included all the comments properly and no more comments.

Is the work clearly and accurately presented and does it cite the current literature?

Partly

If applicable, is the statistical analysis and its interpretation appropriate?

Partly

Are all the source data underlying the results available to ensure full reproducibility?

No source data required

Is the study design appropriate and is the work technically sound?

Yes

Are the conclusions drawn adequately supported by the results?

Yes

Are sufficient details of methods and analysis provided to allow replication by others?

Partly

Reviewer Expertise:

Aquaculture; Immunology; Nutrition

I confirm that I have read this submission and believe that I have an appropriate level of expertise to confirm that it is of an acceptable scientific standard.

# Peer review: Reviewer response for version 2

**DOI:** 10.5256/f1000research.77351.r102340

**Published:** 2022-01-06

**Authors:** Mohammed El Basuini

**Affiliations:** 1 Department of Animal Production, Faculty of Agriculture, Tanta University, Tanta, Egypt

More background is required on the application of fermented plant-based diets in aquaculture.

 A simple paragraph or summary on the type of fish used in terms of importance, production, or normal distribution.

 "fermented medicinal herbs (FMH) in combination with molasses and probiotic drink (Yakult) have been used successfully as dietary supplements on a small number of rural catfish farms in Sumatra, Indonesia, for approximately two years." - Is there any data published?

 The chemical composition, especially the percentage of protein and lipid in the feed provided to fish, must be clarified.

 The absolute weight equation: W0 = average **weight** at the beginning of the study (g). Correct it.

 SGR equation is not correct. The correct formula is SGR = (Ln Wt - Ln W0) / t where:

- SGR = Specific Growth Rate
- Ln = Natural logarithm
- Wt = Weight of fish at the end of the experimental period (T)
- W0 = Weight of fish at the initial of the experimental period
- t = Experimental period in days

Does data meet the assumption of homogeneity of variances? Clarify if the authors ran a homogeneity test. How authors presented their results (Means ± SE or SD).

 Feed utilization (FCR and feed efficiency) is not described in the result section.

 Glucose is an indicator of exposure to stress, as glucose level rises with the level of stress. How do the authors explain the increased glucose level with feed additives?

Is the work clearly and accurately presented and does it cite the current literature?

Partly

If applicable, is the statistical analysis and its interpretation appropriate?

Partly

Are all the source data underlying the results available to ensure full reproducibility?

No source data required

Is the study design appropriate and is the work technically sound?

Yes

Are the conclusions drawn adequately supported by the results?

Yes

Are sufficient details of methods and analysis provided to allow replication by others?

Partly

Reviewer Expertise:

Aquaculture; Immunology; Nutrition

I confirm that I have read this submission and believe that I have an appropriate level of expertise to confirm that it is of an acceptable scientific standard, however I have significant reservations, as outlined above.

# Peer review: Reviewer response for version 1

**DOI:** 10.5256/f1000research.55942.r92787

**Published:** 2021-08-31

**Authors:** Saurav Kumar

**Affiliations:** 1 Aquatic Environment and Health Management Division, ICAR- Central Institute of Fisheries Education, Mumbai, India

**Title: **

- Correct *Pangasianodon *spelling.

**Abstract:**

- Write only significant results and no need to mention values of estimated parameters in this section.

**Introduction: **

- Author needs to highlight the researchable problem with normal herbal immunostimulant, and why is the fermentation process required for preparing the product?

**Methodology: **

- Fish acclimatization and standard procedure of FHM product preparation need to be mentioned with suitable references.
- Statistical analysis of data needs to be mentioned.

**Results:**

- Why do the significant change in the control group observe in RBC of 30 days sampled fish from 60 days?
- Do the experimental periods have a significant effect on observed parameters?
- Similar changes are also observed in haemoglobin, hematocrite values...

**Discussion:**

- The section is well written however the mechanism need to be illustrated.

**References: **

- Very few recent references and authors need to cite the recent findings.

Is the work clearly and accurately presented and does it cite the current literature?

Partly

If applicable, is the statistical analysis and its interpretation appropriate?

No

Are all the source data underlying the results available to ensure full reproducibility?

Partly

Is the study design appropriate and is the work technically sound?

Yes

Are the conclusions drawn adequately supported by the results?

Yes

Are sufficient details of methods and analysis provided to allow replication by others?

Partly

Reviewer Expertise:

Aquatic Environment and Health  Management

I confirm that I have read this submission and believe that I have an appropriate level of expertise to confirm that it is of an acceptable scientific standard, however I have significant reservations, as outlined above.

# Peer review: Reviewer response for version 1

**DOI:** 10.5256/f1000research.55942.r87441

**Published:** 2021-07-22

**Authors:** S. M. Sharifuzzaman

**Affiliations:** 1 Institute of Marine Sciences, University of Chittagong, Chittagong, Bangladesh

**Some specific comments:**

 1) Please revise title as  "Fermented medicinal herbs improve hematological and physiological profiles of Striped catfish *(Pangasi* *onodon* *hypophthalmus)*"

 2) "Some researchers have reported the impact of herbal supplemented diets on hematology and innate immunity of fish" - so why were you combining molasses and probiotics? Please explain this in introduction section.

 3) What were the probiotics cell numbers in different experimental diets?   

 4) An additional experiment with medicinal herbs, i.e. cutchery ( *Kaempferia galanga*), turmeric ( *Curcuma longa*) and curcuma ( *Curcuma zanthorrhiza*) devoid of molasses, probiotic drink (Yakult) and yeast ( *Saccharomyces cerevisiae*) would be interesting to determine the role of probiotics and prebiotics - the authors are requested to include it.

Is the work clearly and accurately presented and does it cite the current literature?

Yes

If applicable, is the statistical analysis and its interpretation appropriate?

Yes

Are all the source data underlying the results available to ensure full reproducibility?

Yes

Is the study design appropriate and is the work technically sound?

Partly

Are the conclusions drawn adequately supported by the results?

Yes

Are sufficient details of methods and analysis provided to allow replication by others?

Partly

Reviewer Expertise:

Aquaculture health and nutrition

I confirm that I have read this submission and believe that I have an appropriate level of expertise to confirm that it is of an acceptable scientific standard, however I have significant reservations, as outlined above.
